# The Microbiome–Estrogen Connection and Breast Cancer Risk

**DOI:** 10.3390/cells8121642

**Published:** 2019-12-15

**Authors:** Sheetal Parida, Dipali Sharma

**Affiliations:** Department of Oncology, Johns Hopkins University School of Medicine and the Sidney Kimmel Comprehensive Cancer Center at Johns Hopkins, Baltimore, MD 21231, USA; sparida1@jhu.edu

**Keywords:** dysbiosis, microbiome, breast cancer, obesity, oral microbiome, hormone regulation, xenobiotics, microbial metabolites

## Abstract

The microbiome is undoubtedly the second genome of the human body and has diverse roles in health and disease. However, translational progress is limited due to the vastness of the microbiome, which accounts for over 3.3 million genes, whose functions are still unclear. Numerous studies in the past decade have demonstrated how microbiome impacts various organ-specific cancers by altering the energy balance of the body, increasing adiposity, synthesizing genotoxins and small signaling molecules, and priming and regulating immune response and metabolism of indigestible dietary components, xenobiotics, and pharmaceuticals. In relation to breast cancer, one of the most prominent roles of the human microbiome is the regulation of steroid hormone metabolism since endogenous estrogens are the most important risk factor in breast cancer development especially in postmenopausal women. Intestinal microbes encode enzymes capable of deconjugating conjugated estrogen metabolites marked for excretion, pushing them back into the enterohepatic circulation in a biologically active form. In addition, the intestinal microbes also break down otherwise indigestible dietary polyphenols to synthesize estrogen-like compounds or estrogen mimics that exhibit varied estrogenic potency. The present account discusses the potential role of gastrointestinal microbiome in breast cancer development by mediating metabolism of steroid hormones and synthesis of biologically active estrogen mimics.

## 1. Background

Humans are not merely individuals but a complex ecosystem harboring trillions of microbial cells. Although microbes inhabit multiple body sites, most of the microbial biomass resides in the gastrointestinal tract. The 100 trillion microbes residing in the GI tract carry a collective genome called the metagenome that is 150 times larger than human genome [1]. Once regarded inert, the dynamic microbiome is now known to accomplish multiple important functions for the human body. The microbiome is undoubtedly the second genome of the human body and sufficient evidence has been accumulated emphasizing its diverse role in health as well as disease. Despite the recognition of the biological impact of the microbiome, translational progress has been limited, partly because the majority of commensals are not conventionally culturable, and partly due to the vastness of the microbiome accounting for over 3.3 million genes [2] whose functions are not fully understood. In addition, microbes reside as consortia in different body sites, and most of the known physiological functions of microbial dysbiosis are community-effects rather than effects of individual microbes. On the bright side, we are now equipped with deep sequencing techniques like shotgun sequencing and multiple tools to perform gene and functional annotations enabling better understanding of the genome–metagenome puzzle. The findings of the human microbiome project continue to unearth the complex metabolic interactions of the microbiome with the genome and it is no surprise that dysbiosis can induce and aid multiple organ-specific cancers including colorectal, stomach, anogenital, hepatic, pancreatic, prostrate, and breast cancer. The microbiome aids carcinogenesis by (i) stimulating host cells to proliferate or affecting programmed cell death, (ii) priming and regulating the immune response, and (iii) altering metabolism of indigestible dietary components, xenobiotics, and pharmaceuticals [3,4]. The etiology of breast cancer is complex and multiple risk factors have been described for different subtypes of breast cancer. Interestingly, genetics account for less than 10% of breast cancers and as many as 70% of breast cancers occur in women at seemingly-average risk [1]. While the biological reasons remain poorly understood, approximately 70% of all breast cancers are estrogen receptor positive subtype. Most of these ER positive breast cancers are detected in post-menopausal women where systemic estrogen levels have already declined and estrogen is produced primarily by adipose tissue and via the aromatization of androgen precursors [5]. Of note, one of the vital functions of the microbiome is regulating the levels of steroid hormones in the body, and—most importantly—estrogen. The regulation of estrogen metabolism by intestinal microbes has been known for over a decade now and yet our understanding in this regard is limited due to the complexity of the microbiome that can vary based on diet, BMI, ethnicity, race, age, occupation, disease status, and antibiotic usage. This has resulted in inconsistent outcomes in epidemiological studies. In this review, we discuss the regulation of estrogen metabolism by commensals and its potential role in breast cancer.

## 2. Dysregulated Gut Microbiome Is Observed in Women with Breast Cancer

An association between altered gut microbiome and breast cancer was reported as early as 1990 in a study comparing the gut microbiome of 7 healthy women and 11 women with breast cancer. This study found that *Clostridia*, *Enterobacterium*, *Lactobacilli*, *Bacteroides*, and *Escherichia coli* were enriched in breast cancer patients [6]. Recently, gut microbial diversity was examined in 18 premenopausal controls, 25 premenopausal women with breast cancer, 44 postmenopausal controls, and 46 postmenopausal women with breast cancer [7]. This study utilized shotgun metagenomic analysis allowing detailed taxonomic classification and functional analysis as opposed to earlier studies that relied on 16srRNA sequencing and biochemical characterization. Women with breast cancer exhibited more diverse gut microbiome in comparison to controls. Variation between premenopausal cases and controls was marginal whereas in postmenopausal women, both species richness and species diversity were higher in women with breast cancer; *p* = 0.003 and < 0.001 respectively. At least 45 species showed significant differential abundance between cases and controls in postmenopausal cohort (listed in Table 1). Using a random forest classifier, they reported 14 potential microbial markers for postmenopausal women with breast cancer including *Fusobacterium varium*, *Shigella_sp_D9*, *Desulfovibrio piger, Escherichia_sp_1_1_43*, *Shigella sonnei, Eubacterium eligens*, *Escherichia_sp_3_2_53FAA*, *Vibrio cholerae, Acinetobacter baumannii*, *Proteus mirabilis*, *Fusobacterium nucleatum*, *Campylobacter concisus*, *Escherichia coli*, and *Porphyromonas uenonis* with remarkable sensitivity and specificity. Gene annotation showed a higher abundance of pathogen–host interaction genes (*p = 0.021*) as well as virulence factors (*p* = 0.016) in postmenopausal women with breast cancer compared to controls. Virulence factors encoded were LOS glycosyltransferase, peritrichous flagella, and type I fimbriae. Association of gut microbes with clinical indices were examined and *Acinetobacter radioresistens* and *Vibrio cholerae* correlated positively but weakly while Yersinia enterocolitica correlated negatively with C4 levels. Acinetobacter radioresistens and *Enterococcus gallinarum* showed weak positive correlation to high-sensitivity C-reactive protein expression while *Shewanella putrefaciens* and *Erwinia amylovora* exhibited weak positive association with estradiol levels. *Porphyromonas uenonis* showed weak positive and Enterococcus gallinarum showed weak negative correlation with CD19. Actinomyces sp. *HPA0247* was negatively proportional to CD3^+^CD8^+^ T cell numbers, though the correlation was weak. Metagenome of postmenopausal women with breast cancer showed enrichment of lipopolysaccharide biosynthesis, iron complex transport system, vitamin B12 transport system, PTS system, secretion system, and beta-oxidation genes. Butyrate synthesis was diminished in postmenopausal women with breast cancer. In intestinal epithelial cells butyrate inhibits NF-κB activation preventing inflammation and it is also known to induce immune cell activation [8]. Similar to some earlier studies, dysbiosis was found to be associated with postmenopausal but not premenopausal breast cancer [7]. Enteric infection with *H. hepaticus* has also been shown to increase incidence of mammary tumors in C57BL/6 ApcMin Rag2 deficient mice, though the model used is not deemed suitable [9]. Overall, several key changes have been observed in gut microbiome of women with breast cancer suggesting a strong link between microbial dysbiosis and breast cancer.

## 3. Gut Microbiome, Estrobolome, and Breast Cancer Connection

**Figure 1:**
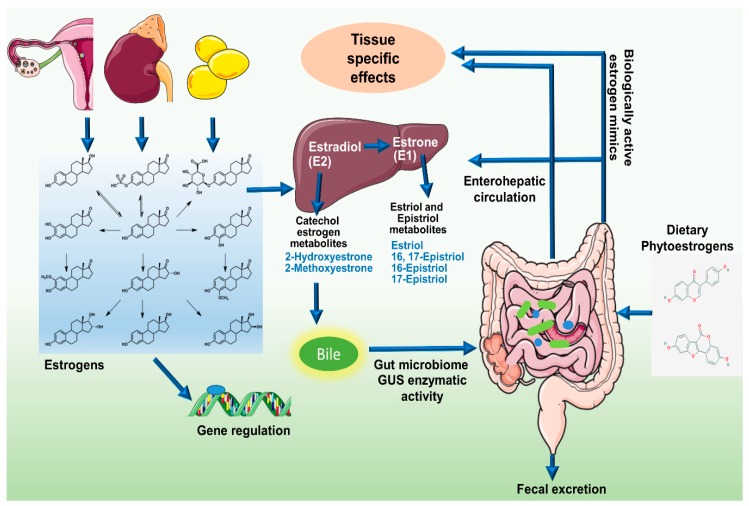
Schematic representation of modulation of estrogens and its metabolites in circulation by the estrobolome. The C-18 steroid hormones, Estrogens (E1, E2, and E3) circulate in the blood stream either in free or protein bound form exerting diverse biological effects. Hepatic metabolism of parent estrogens E2 and E1 irreversibly hydroxylates the C2, C4, or C16 positions of the steroid ring producing estrogen metabolites with varying hormone potency, bioavailability, and half-life. Estrogens and their metabolites are then conjugated in the liver through glucuronidation and sulfonation to allow biliary excretion. While most of the conjugated estrogens are excreted in urine or feces, a significant proportion is reabsorbed into the circulation. Gut bacteria possessing β-glucuronidase activity can deconjugate the conjugated estrogens leading to reabsorption into the circulation. In addition, enteric microbes synthesize estrogen-like compounds or estrogen mimics from dietary sources.

Endogenous estrogen is undoubtedly a major player in breast cancer, especially after menopause, since 70% of breast cancers belong to estrogen-receptor positive subtype. Before menopause, the primary site of estrogen synthesis is the ovaries and circulating estrogen acts on multiple target sites including the bones, brain, and immune system in an endocrine fashion [10]. However, in postmenopausal women, estrogen production is extragonadal, mainly in adipose tissue, bones, and the brain, functioning in a paracrine or intracrine fashion [10]. Local estrogen levels are regulated by tissue specific CYP19 expression [10]. In breast cancer patients, CYP19 and aromatase expressions are elevated in breast adipose tissue up to 20-folds more than the circulating levels [10]. The stimulus for enhanced estrogen biosynthesis comes from tumor derived factors like prostaglandins secreted by fibroblasts, infiltrating lymphocytes or even epithelial cells [10]. Estrogens are C-18 steroid hormones, derived by multistep cholesterol (C27) reduction. Endogenous estrogens exist in three biologically active forms, estradiol (E2) (premenopausal), estrone (E1) (postmenopausal), and estriol (E3) (in pregnant women) [11,12]. Estrogens can circulate in the blood stream as free moieties or in protein-bound form and vary in their potency and biological significance [12]. In the liver, parent estrogens E2 and E1 irreversibly hydroxylate at C2, C4, or C16 positions of the steroid ring [12]. Respective metabolites have been indicated in Figure 1. These estrogen metabolites possess different hormone potency, bioavailability and half-life [12]. They are conjugated via glucuronidation and sulfonation and prepared for biliary excretion [11,12,13]. In experimental animals injected with radiolabeled hormones, 65% of E2, 48% E1, and 23% of E3 were recovered in the bile [12]; 10% to 15% were excreted in conjugated form in feces and the remaining amount of estrogens were reabsorbed into the circulation [12]. It is important to note that regulation of estrogen levels in the body is largely dependent on the microbiome [11,12,13,14,15].

The sum total of bacterial genes in the gastrointestinal tract capable of metabolizing estrogens has been termed as “estrobolome” by Plottel and Blaser [11]. An active estrobolome is thought to modulate the endogenous estrogen metabolism via the enterohepatic circulation by virtue of bacterial β-glucuronidases and β-glucosidases enzyme activity, therefore affecting circulating and excretory estrogen levels [14]. The enterohepatic circulation of estrogens, their conjugation and deconjugation by enteric microbes and reabsorption into the circulation is schematically presented in Figure 1. Most gut bacteria exhibit β-glucuronidase enzyme activity that enables them to deconjugate the conjugated estrogens leading to their reabsorption into the circulation [14,16,17]. In human GI tract, the most important β-glucuronidase encoding genes are the GUS genes. Mammalian UDP-glucosyltransferases link glucuronic acid moiety to complex compounds including steroid hormones marking it for excretion [14]. Intestinal microbes possessing GUS genes encoded β-glucuronidase enzymatic activity can remove the glucuronic acid to be used as carbon source. The respective aglycones are either released into the GI tract for excretion or released back into the circulation [14]. The enterohepatic circulation similarly metabolizes an array of complex molecules including neurotransmitters, anti-cancer agents, non-steroidal anti-inflammatory drugs (NSAIDs), and environmental carcinogens and marks them for excretion but whether they are excreted or recycled back into the circulation is largely determined by the gut bacteria [2,17]. Recently, an atlas of β-glucorunidases in human GI tract microbe has been characterized. Approximately, 112 novel GUSs have been identified and clustered into 6 classes expressed in 4 bacterial phyla, namely, Bacteroidetes, Firmicutes, Verrucomicrobia, and Proteobacteria [17]. Among them phylum Bacteroidetes presents highest abundance and diversity of GUS enzymes [17].

Interestingly, β-glucuronidase activity gets modulated depending on diet and bacterial context [11,12,14,16]. Phylogenetically, 80% of an individual’s microbiome is composed of *Firmicutes* and *Bacteroidetes* followed by minor phyla including Proteobacteria, Actinobacteria, *Verrucomicrobia*, Cyanobacteria, and Tenericutes. A lower ratio of *Firmicutes* to *Bacteroidetes* is usually associated with a healthy state. A diet rich in fat or protein has been found to be associated with higher fecal β-glucuronidase activity whereas fiber consumption decreases its activity [12]. In addition, β-glucuronidase activity of cultured *Escherichia coli* is known to be governed by population density, thereby suggesting the involvement of quorum sensing in controlling the enzymatic activity [12,18]. Adding to the complexity, the hepatic sulfatase pathway converts inactive steroids in circulation to active estrogens [11,12]. Sulfatase positive gut bacteria hydrolyze estrogen molecules excreted into the GI tract after hepatic sulfation. Whether bacterial β-glucuronidase and β-glucosidase contribute to breast cancer risk remains to be determined; accumulating evidence, as discussed in this section, suggest a potential role. Hydroxysteroid dehydrogenases is another enzyme encoded by certain classes of gut bacteria [19,20]. Hydroxysteroid dehydrogenases are basically alcohol oxidoreductases [12] which partially catalyze estrogen biosynthesis from cholesterol [19,20]. Hydroxysteroid dehydrogenase enrichment may modulate the ratios of conjugated estrogens as well as the metabolism of androgens, shaping the hormonal environment of the host.

## 4. An Active Estrobolome Regulates Estrogen Levels

**Figure 2:**
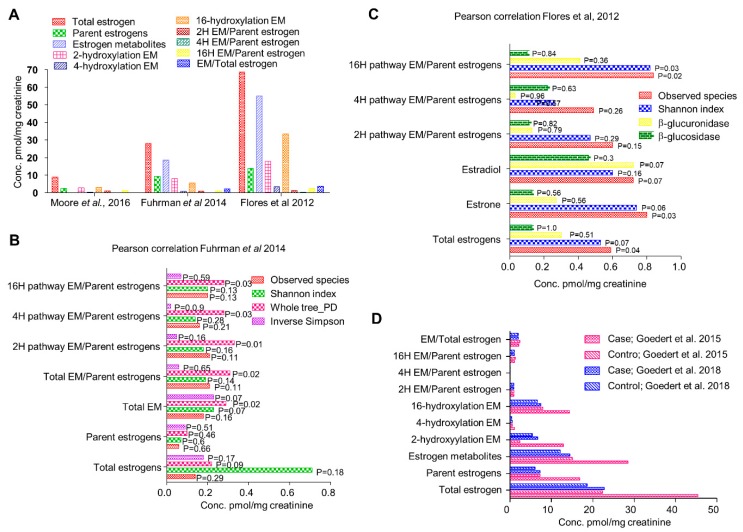
(**A**) Relative circulatory levels of parent estrogens and its metabolites in different cohorts (data obtained from respective studies as indicated). (**B**) Correlation of systemic estrogen and estrogen metabolites microbiome diversity (analyses of data published by Fuhrman et al., 2014). (**C**) Correlation of systemic estrogen and estrogen metabolites microbiome diversity (analyses of data published by Flores et al., 2012). (**D**) Comparison of relative circulatory levels of parent estrogens and its metabolites in two different cohorts (Data obtained from respective studies).

Over the decades, many studies have supported the estrobolome hypothesis (Figure 2) [12,13,14,15,16,19,21,22,23,24,25,26,27,28,29,30,31,32,33,34]. Germfree rats excrete unquantifiable amounts of hormones whereas conventional rats, under the same conditions, excrete significant amount of free steroid hormones measurable by gas chromatography and mass spectrometry [33]. Introduction of bacteria normalizes the estrous cycle of infertile germfree female mice and increases the sperm counts in male mice restoring fertility [33]. Fuhrman et al. demonstrated significant positive correlation between the ratio of estrogen metabolites to parent estrogens and phylogenetic diversity of gut microbiome [21]. Gut microflora varies with race, ethnicity, diet, BMI, antibiotic exposure, and infections [26,29,35,36,37,38] and plays an important role in driving hormone dependent cancers by catalyzing deconjugation and reabsorption of estrogen [5,23,24]. Antibiotics perturb gut microbial populations and a number of studies have shown a decrease in excretion of conjugated steroid hormones upon administration of common antibiotics like ampicillin and oxytetracycline in both men and women [34,39,40]. Antibiotics reduce fecal β-glucuronidase activity in rats [12] suggesting at least short-term effect of antibiotic exposure on circulatory levels of steroid hormones; long-term effects remain elusive. Some epidemiologic studies suggest positive correlation between antibiotic use and breast cancer risk [24,39]. In a North American case control study, long term antibiotic treatment associated positively with increased breast cancer risk (Odds ratio 2.07) [23]. Breast cancer risk enhanced irrespective of class of antibiotic, age, menstrual status, or family history [23]. A marginal increase in breast cancer risk with antibiotic exposure was also observed in a study with 2.1 million women followed over a period of nine years; hazard ratio 1.14 (95% CI ≥ 1.1 to 1.18), but little dose response was observed with long term antibiotic usage; hazard ratio of 1.17 (95% CI ≥ 0.97 to 1.42) [39]. In a population-based study encompassing 3,112,624 women, risk of breast cancer increased slightly but positively with prolonged antibiotic usage when adjusted for BMI, smoking, diabetes and hormone replacement therapy [41]. Though these studies suggest that prolonged antibiotic use only moderately increases breast cancer risk, the actual magnitude could be much higher since a large proportion of antibiotics remain unaccounted.

Since initial microbiome is established at birth, even exposure during infancy can potentially impose a lifelong impact [36,42]. Gut microbial composition and hormone metabolism are largely diet-dependent. In population-based studies, vegetarians have been found to excrete more conjugated estrogens compared to their non-vegetarian counterparts, probably resulting in lower levels of estrogens in plasma. In an American study involving 10 premenopausal women, vegetarians had 3-fold higher levels of conjugated estrogens in feces and the plasma estrogen levels were 15% to 20% lower compared to omnivores as was the fecal bacterial β-glucuronidase activity [12,43]. In healthy postmenopausal women, alcohol consumption also correlated with significantly higher plasma E1 and to a lower extent, E2 concentrations [12]. This could be correlated with the fact that small intestinal bacterial overgrowth (SIBO) can result from higher alcohol consumption as observed in animal models of alcoholic liver disease [12] and may alter microbial metabolic pathways, though further studies are required. In experimental rats, chronic ethanol consumption altered the fatty acid, bile acid, amino acids and 4-hydroxyestrone metabolism [44]. 4-hydroxyestrone is a catechol estrogen metabolite, a biologically active estrogen; it has been shown to induce estrogenic tumorigenesis in animal models [12,44]. Oral consumption of *Lactobacillus acidophilus*, induced a decrease in fecal enzyme activity including β-glucuronidase [12].

Flores et al., in 2012, investigated the systemic and fecal levels of estrogen and its 13 metabolites and their association with gut microflora in a group consisting 25 men, 7 postmenopausal women and 19 premenopausal women (Figure 2A) [15]. Fecal β-glucuronidase and β-glucosidase enzymatic activities were quantified by real time kinetics; microbial diversity was estimated by 16srRNA pyrosequencing and estrogen and its metabolites in urine and feces were determined by liquid chromatography/tandem mass spectrometry (LC/MS) [15]. In both men and postmenopausal women where estrogen is non-ovarian, levels of total urinary estrogen and most of the estrogen metabolites showed strong correlation with richness and alpha diversity of fecal microbiota (R ≥ 0.5, *p* ≤ 0.003) (Figure 2C). Multivariable adjustment for BMI, age and sex had negligible effect on species richness and alpha diversity. However, no correlation in any of the group was observed with respect to β diversity. They also observed significantly strong association of systemic estrogens with fecal taxa *Clostridia* in *Firmicutes* and genera from *Ruminococcaceae* family (β = 0.57 to 0.7, *p =* 0.03 to 0.002) [15]. Fecal β-glucuronidase was proportional to urinary estrone concentration (R = 0.36, *p =* 0.04) but not to other estrogen metabolites [15]. In contrast, it was negatively proportional to total estrogen in the feces. In premenopausal women, no correlation of any kind was observed, possibly because they were all in different phases of the menstrual cycle. This study suggests that fecal β-glucuronidase is negatively proportional to the total estrogen levels in circulation and positively proportional to total urine estrone level [15]. Microbial richness in the feces were directly and strongly associated with total systemic estrogens but independent of any phylogenetic class or cluster. Taxa *Clostridia* of phylum *Firmicutes* showed significant correlations and its functional and metabolic correlations should be further investigated.

Another study in 2014 by Fuhrman et al. investigated the association of fecal microbiome with estrogen and its metabolites in urine of postmenopausal women [21]. In a group of 60 healthy postmenopausal women not undergoing any hormone replacement therapy or antibiotic regimen, they checked the creatinine-standardized levels of estrogens and its metabolites by liquid chromatography-tandem mass spectrometry. Phylogenetic composition of fecal microbiota was assessed by 16srRNA pyrosequencing and general linear models were used to find correlation with conjugated and non-conjugated estrogen levels (Figure 2A). The ratio of estrogen metabolites to their precursors was found to be directly proportional to the whole tree phylogenetic diversity (R = 0.35, P = 0.01) [21]. Interestingly, as observed in earlier study, ratio of metabolites to their precursors was proportional to the abundance of order *Clostridiales* (R = 0.32, *p =* 0.02) (Figure 2B). Similar correlation was also observed with abundance of genus *Bacteriodes* (R = −0.3, *p =* 0.03) [21] (Figure 2B). In addition, the associations were independent of age, BMI and study design factors suggesting that women with a more diverse microbial ecosystem in their gut exhibit a higher ratio of estrogen metabolite to their precursors in urine. Parent estrogens formed 32% of total estrogen while 2, 4, and 16 hydroxylated metabolites represented 29%, 3%, and 35% of total estrogen metabolites [21]. Total estrogen metabolites varied with BMI; underweight/normal < overweight < obese, *p =* 0.11 [21]. Contrary to conventional knowledge that microbial populations modulate systemic estrogen levels by deconjugating estrogen metabolites secreted in the bile, the estrogen metabolites exhibited stronger correlation with species diversity compared to parent estrogens suggesting that microbial population may differentially affect the components of total estrogen metabolites. One way to investigate the association would be to pharmacologically inhibit bacterial β-glucuronidase activity. This study confirms that in random group of postmenopausal women, gut microbial diversity is positively associated with ratio of estrogen metabolites to parent estrogens.

Further in 2015, Goedert et al. investigated the differences in gut microbial population of 48 postmenopausal women with breast cancer and same number of age matched healthy women [25] (Figure 2D). Surprisingly, the total estrogens in urine directly correlated with α-diversity in the control group (Spearman Rho = 0.37, *p =* 0.009) but not in cancer cases (Spearman Rho = 0.04, *p =* 0.77) [25]. Cancer cases had significantly altered species diversity compared to controls (β-diversity, *p* = 0.006) as well as lower α-diversity, *p =* 0.004. All mean estrogens were 2-fold higher in cancer cases as compared to controls, but remained statistically insignificant, *p* ≥ 0.1 [25]. In control women, fecal whole tree phylogenetic diversity correlated with total estrogens but not in women with breast cancer. Ratio of estrogen metabolite to parent estrogen showed weak positive correlation with alpha diversity in controls (Spearman Rho = 0.26, *p =* 0.08) but not in cancer cases (Rho = −0.11, *p =* 0.45). Cancer cases exhibited lower alpha diversity in fecal microbiota (*p* ≤ 0.004) except Shannon index, *p =* 0.09 [25]. Microbiota composition also varied across all genus level taxa. Specifically, in order *Clostridiales*, cancer cases carried higher levels of *Ruminococcaceae*, *Clostridiacae* and *Faecalibacterium* but lower levels of *Dorea* and *Lachnospiraceae* [25]. In 2016, Moore et al. analyzed the levels of estrogen and its 13 metabolites in 399 postmenopausal invasive breast cancer patients against 399 age matched controls from Shanghai, China [13] (Figure 2A). A very strong correlation of urinary estrogen with breast cancer risk was observed (OR = 1.94, 95% CI = 1.21 to 3.12, *p*_trend_ = 0.28). The ratio of 2-pathway metabolites to parent estrogens as well as total estrogens inversely correlated with breast cancer risk. Comparing the levels of urinary estrogens and estrogen metabolites, it was three-fold higher in Asian American women compared to women from Shanghai. Concentrations of 2-pathway and 16-pathway metabolites were found to be similar to the parent estrogens (31% and 36% against 29%) [13]. The 2 and 4 pathway ratios strongly correlated with each other but not with 16 pathway hydroxylation products [13]. Breast cancer risk was strongly associated with the urine concentrations of total estrogens, parent estrogens and the 16 hydroxylation pathway products. Though lower in magnitude, breast cancer risk was positively correlated with 2 and 4 pathway metabolites [13]. Breast cancer risk was found to be inversely proportional to ratio of 2-pathway metabolite to total estrogens and to parent estrogens. In population based comparison, Asian American females had three-fold higher circulating estradiol, twice the levels of total estrogens and 4 pathway metabolites and the differences were still significant when adjusted for BMI [13]. These differences were consistent with the fact that other breast cancer studies saw ~2.5 folds higher levels of circulating estradiol levels in women from high-risk countries compared to low-risk nations like China. BMI has been regarded as a primary factor determining circulating estrogen levels. However, prior studies report only 15–16% variance in estradiol levels that could be explained by BMI [45,46,47]. We therefore presume that other factors might be responsible for these wide differences; one attributable factor being the microbiota.

## 5. Enteric Microbes and Phytoestrogens

In addition to metabolizing estrogens, enteric microbes perform a very important role in synthesis of estrogen-like compounds or estrogen mimics from dietary sources [27,32,48,49,50,51,52]. Phytoestrogens are group of plant compounds containing one to two benzene ring and two or three hydroxyl groups, similar to 17β-estradiol in structure and molecular weight [53]. Owing to structural similarities, they are capable of binding to estrogen receptors ERα and ERβ. These compounds include isoflavanoids, flavonoids, lignans, coumestans, ellagitannins, stilbenes [53], genistein, daidzein and its metabolite S-equol, and coumestrol [54]. Only a few of them possess actual estrogenic or anti-estrogenic activities. S-equol, one of the most potent phytoestrogen is exclusively produced by intestinal microflora [55]. Affinity of ERs for phytoestrogens is about 4 folds lower than 17β-estradiol, but with dietary intake of precursors reaching 10 to 100 mg per day, the circulating concentrations could reach micromolar levels, hence impact could be significant [55]. Interestingly, phytoestrogens have much higher affinity for ERβ compared to ERα, which might be of evolutionary significance since estrogen dependent breast cancers are usually mediated by ERα while other physiological actions are mediated by ERβ. Of note, isoflavones have several times higher affinity for ERβ than for ERα, therefore some selectivity in physiological activity among phytoestrogens can be expected [56]. However, some triple negative breast cancers exhibit active ERβ [57]. Some phytoestrogens interfere with steroid hormones biosynthesis; for example, dehydrogenases of 17β-hydroxysteroids, 3β-hydroxysteroids, and aromatase lowering testosterone and estradiol production [55]. They also influence bioavailability by inhibiting sulfatase and sulfotransferase [55]. Polyphenols like Isoflavones and lignans are present in plant sources as glycosides with methylated hydroxyl groups [55]. Intestinal bacteria like *Eubacterium limosum*, demethylate the hydroxyl group activating the estrogenic compounds [55]. Biotransformation of these compounds in humans is carried out by *Adlercreutzia equolifaciens*, *Eggerthella spp*., and *Slackia isoflavoniconvertens* [55].

**Figure 3:**
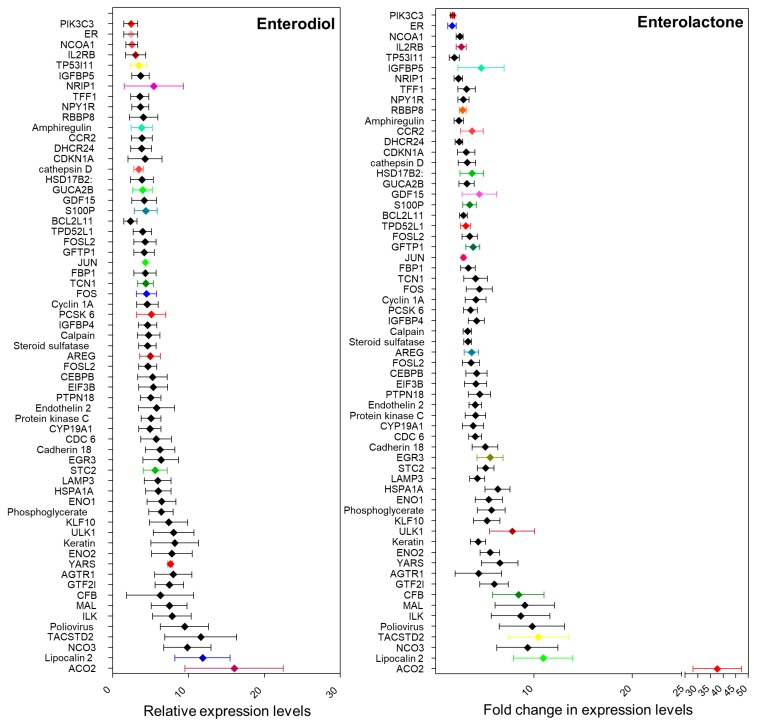
Relative expression levels of estrogen responsive genes in MCF7 cells treated with Enterodiol and enterolactone (Analyses of GEO2R, [32]).

Plant-based lignans are present in high concentrations in soy, flax seeds, and sesame, and to some extent in fruits, vegetables, and berries [52]. These lignans are converted into compounds like enterodiol, enterolactone, sesamin, matairesinol, and pinoresinol [27,32]. Enterolactone is produced by the action of *Eggerthella* [55]. The debate on the effects of these compounds on ER positive breast cancer risk is more than a decade old. It has been suggested that phytoestrogens can modulate estrogen metabolism by inhibiting aromatase, therefore reducing levels of circulatory estrogens [32]. It is also thought to competitively bind to ER owing to its structural similarity with estrogen, thus reducing its bioavailability [32]. Multiple studies have been conducted to examine the serum levels of these compounds in different populations [27,32,51,52]. Most of these studies have been inconclusive due to huge variations in the serum levels of these compounds as well as appreciable differences in dietary habits. Moreover, the actual source of these compounds and the metagenomic composition of the individuals have never been considered. A comprehensive meta-analysis comparing epidemiologic data from all population-based studies found a slight decrease in breast cancer risk in post-menopausal women with high levels of serum enterolignans [52]. However, some cell-based studies using ER positive cell lines suggest an estrogenic effect that might lead to an increase in breast cancer risk. We analyzed one such microarray dataset (GSE86565) available on NCBI-GEO where MCF7 cells were treated with enterodiol, enterolactone, and their precursors seasmin, matairesinol, and pinoresinol [32]. Alteration in the expression level of estrogen responsive genes was examined (Figure 3). Enterolactone is the most prevalent phytoestrogen. Similar to estrogen, enterolactone, synthesized in the gut by metabolism of dietary lignans by the microbes, can be absorbed in the gut and enter the enterohepatic circulation [58]. Antibiotics have been found to inhibit enterolactone synthesis for about a year suggesting that gut microbiome altered by external agents could take a year or longer before it can recover [41].

Multiple studies have been conducted to examine the role of enterolignans in cancer incidence and progression [58]. While some epidemiologic studies project them as potential anti-proliferative agents for breast cancer [59], many other reports suggest otherwise. In a recent meta-analysis, serum enterolactone was found to be protective against breast cancer, predominantly, in postmenopausal women [60]. Similarly, in the animal experiments, synthesis of enterolignans from dietary lignans by gut microbes has a protective effect on breast cancer [61]. But enterolignans can also activate the estrogen receptor transcription machinery [62]. A cross-sectional mammogram study showed that enterolactone level slightly positively correlated with an increase in mammographic density (*p* < 0.01) [62]. Interestingly, serum enterolactone level is inversely proportional to obesity, yet another breast cancer risk factor [63]. As such, obesity and high BMI are negatively proportional to gastrointestinal microbiome diversity, hence lower the BMI higher the rate of microbial metabolism [38]. In postmenopausal women, body fat percentage in proportional to the levels of parent estrogens and 2-hydroxylation metabolites ratio [21], suggesting a diminished microbial fermentation activity in the gut and thus lower microbial diversity. A diet rich in fat induces increased bile production to aid its digestion. Bile acids are metabolized by intestinal microbes producing secondary bile acids (SBAs) that are considered potent signaling molecules. SBAs favor the overgrowth of *Proteobacteria* species such as *E. coli*, Klebsiella, Enterobacter, and *Citrobacter* [9]. All these microbes are known to encode beta-glucuronidases, capable of deconjugating estrogens, thus, adding to the estrogenic burden [9].

In addition to estrogen, at least two other steroid pathways have been implicated in breast cancer and are currently being investigated as therapeutic targets. In ER positive breast cancers, androgen receptor (AR) and androgenic enzymes are known to antagonize ERα, suppressing carcinoma progression. AR may also play an important role in luminal androgen receptor (LAR) subtype of triple negative breast cancer. These steroids are thought to aid in stroma activation necessary for breast cancer progression [64]. Neither the alternative steroids nor their association with the microbiome has been studied in detail, however, given the overall role of microbiome in steroid metabolism, it might be relevant in context of breast cancer progression [65].

## 6. Phytoestrogens: To Be or Not to Be Estrogenic

Structural similarities between phytoestrogens and estrogens were discovered in 1980s [66] and competitive binding of these structurally similar compounds to estrogen receptors was thought to be responsible for their estrogenic/anti-estrogenic effects which varies depending on cell lines [66]. As early as 1987, Welshons et al. reported that enterolactone (ENL) induced proliferation of ERα positive breast cancer cell lines MCF7 and T47D at a concentration of 1 μM [67]. In 1999, Wang et al. showed that 10 μM ENL enhanced estrogen induced DNA synthesis but inhibited the same at higher concentrations indicating dose-dependent effects in the presence of estrogen [68]. Similarly, Mousavi et al. demonstrated that ENL induced proliferation in MCF7 cells with concentrations ranging from 0.5 μmol/L to 2 μmol/L but an inhibitory effect was observed with 10 μmol/L ENL. ENL induced-proliferation was reversed in the presence of estrogen [69]. More recently, Potier and group demonstrated a differential ERα transactivation by enterodiol (END) and enterolactone (ENL) in breast cancer cells [70]. They showed that END induces ERα transcriptional activity via AF-1 and AF-2 transactivation while ENL functions mainly via AF-2 transactivation and is more efficient in activating ERβ [70]. These results suggest that END’s effect on breast cancer is similar to estrogen at concentrations 2–3 folds higher than E2 [70]. However, such high circulating concentrations of phytoestrogens are easily achievable with a lignan rich diet under normal physiological conditions. In a recent study by Zhu et al., it was reported that of all enterolignans and their precursors, only ENL induced significant proliferation of MCF7 cells similar to estrogen which could be inhibited by ICI [32]. Using a DNA microarray, they examined gene expression profiles in response to the enterolignans and their precursors. Comparing expression levels of estrogen responsive gene, END and ENL showed high levels of similarities with estrogen [32]. They concluded that although the enterolignans as well as their precursors have estrogenic activity, only ENL is functionally similar to estrogen. On the contrary, Briese and group reported an inhibitory effect of lignans on ER positive (MCF7) and ER negative (BT20) breast cancer cells [71], though the concentration of lignans used was higher than other studies. Saberian and colleagues reported that at 100 μM, both END and ENL reduce cell viability while ENL reduces telomerase activity and inhibits hTERT [72]. Brooks et al. demonstrated that both END and ENL inhibited local estrogen biosynthesis in MCF7 cells. 10 μM END or ENL significantly diminished E1(estrone) production via aromatization while 50 μM ENL alone inhibited E1 to E2 conversion via 17β-hydroxysteroid dehydrogenase [73]. Overall, different studies have put forth discrepant results regarding the biological activity of phytoestrogens in breast cancer, which encouraged us to revisit this question.

## 7. Our Quest For Answers: Are Enterodiol and Enterolactone Estrogenic?

In an effort to understand the microbiome–estrogen connection and its effect on breast cancer risk, we decided to investigate the functional impact of enterolignans on estrogen receptor positive breast cancer cells. Current knowledge regarding the role of enterolignans and other phytoestrogens in breast cancer is discordant but we have to appreciate that the impact of enterolignans may be context specific and not uniform. We hypothesize that enterolignans are potent estrogenic compounds, however their circulating levels can vary dramatically depending on dietary habits, age, menstrual status, and most importantly the gut microbiome composition. Moreover, the levels of enterolignans are variable throughout life and only long-term exposure to high levels of these compounds can cumulate to a significant biological outcome. Tissue specific effects can again vary depending on total estrogen bioavailability and metabolic efficiency. We utilized in silico approaches to predict potential targets of enterodiol (END) and enterolactone (ENL) and their binding efficiency to estrogen receptor (ER) followed by in vitro functional assays to evaluate their effects on ER positive breast cancer cells.

**Figure 4:**
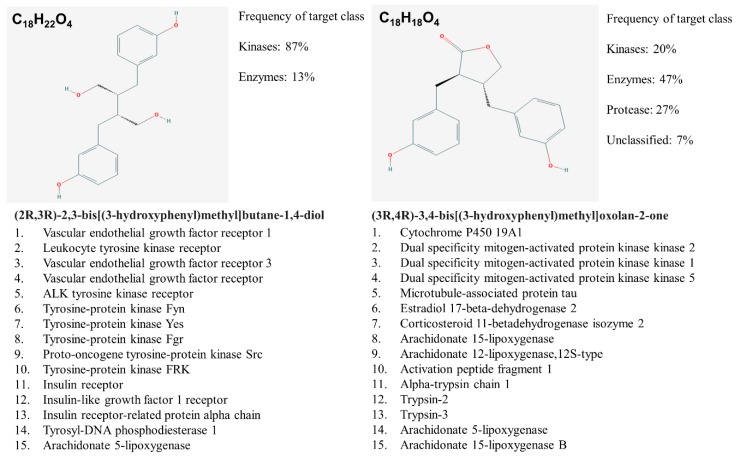
END and ENL biological target prediction using Swiss Dock.

In silico target prediction using SwissDock target scan suggested that the enterolignans can act as ligands to multiple targets including kinases and their receptors in addition to estrogen receptors (ERs) (Figure 4).

**Figure 5:**
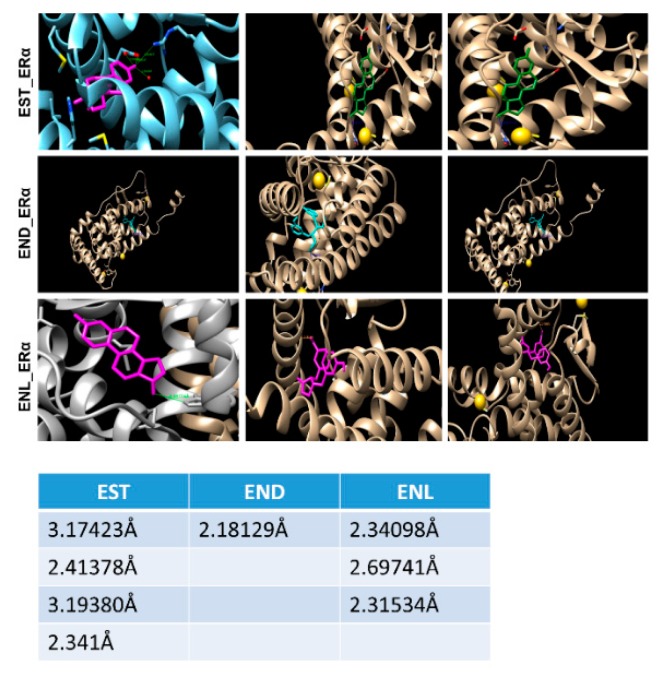
A simulation approach showing binding of Enterodiol and Enterolactone to ERα at site similar to E2. Docking performed using UCSF chimera and AutoDock Vina. Table showing length of hydrogen bonds formed between the respective ligands with ERα.

**Figure 6:**
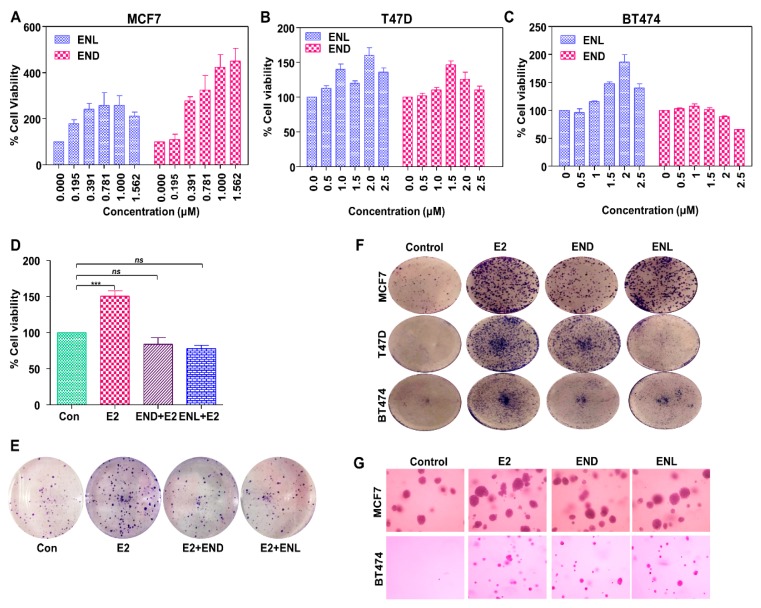
Enterodiol (END) and enterolactone (ENL) enhance proliferation and colony formation in ER positive breast cancer cell lines. (**A**–**C**) MCF7, T47D, and BT474 cells were treated with various doses of END and ENL followed by MTT assay. (**D**,**E**) MCF7 cells were treated with 1 μM END and 1 μM ENL in the presence of 100 nM E2 followed by MTT and clonogenicity assay. (**F**,**G**) BT474, T47D and MCF7 cells were treated with 1 μM END, 1 μM ENL and 100 nM E2 followed by anchorage-independent as well as anchorage-dependent assays.*** *p* < 0.0005; ^ns^ *p* > 0.05.

Molecular docking using AutoDock Vina and UCSF Chimera showed that both END and ENL can bind to ERα and ERβ similar to estrogen (E2) (Figure 5). Next, we examined the effect of END and ENL treatment on cell viability, anchorage-dependent and anchorage-independent growth of breast cancer cells. END and ENL treatment induced cell proliferation in ER positive breast cancer cell lines, MCF7, BT474, and T47D (Figure 6A–C). Interestingly, growth stimulatory effect of END and ENL was apparent in the absence of estrogen (Figure 6A–C) whereas moderate inhibition was observed when END and ENL were used in combination with 100 nM E2 (Figure 6D,E) suggesting that competition for the ligand binding site in the presence of E2 negates their estrogenic activity. END and ENL treatment stimulated clonogenicity and soft-agar colony formation of breast cancer cells (Figure 6F,G). These results showed that breast cancer cells treated with END and ENL exhibit increased cell viability and clonogenic potential. Estrogen binds to estrogen receptor starting a cascade of signaling events resulting in an increased expression of E2-responsive genes.

**Figure 7:**
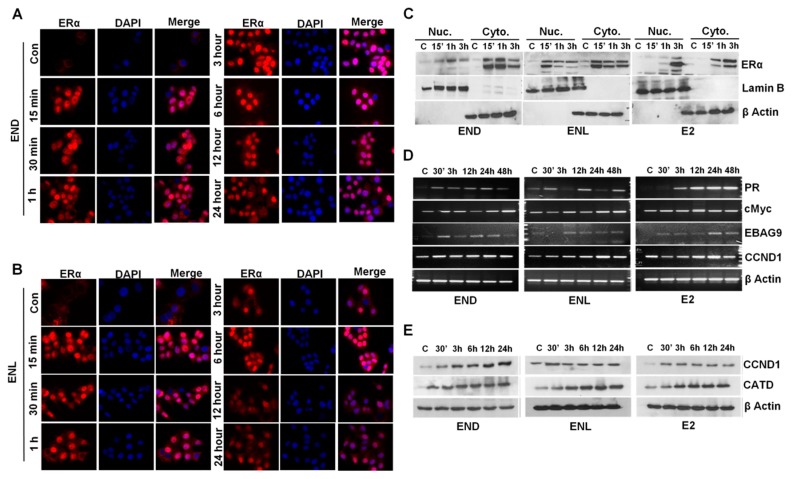
END and ENL induce nuclear translocation of estrogen receptor (ER) and increase the expression of ER-responsive genes. (**A**,**B**) MCF7 cells were treated with 1 μM END and 1 μM ENL for different time intervals as indicated and localization of ER was examined using immunocytochemistry. (**C**) MCF7 cells were treated with 1 μM END, 1 μM ENL, and 100 nM E2 followed by nuclear and cytoplasmic extraction and immunoblot analyses for ER expression. (**D**,**E**) MCF7 cells were treated with 1 μM END, 1 μM ENL, and 100 nM E2 for indicated time intervals. Protein lysates and total RNA were isolated and examined for the expression of ER-responsive genes as noted.

Breast cancer cells treated with END and ENL exhibited nuclear translocation of estrogen receptor within 15 min post-treatment that was diminished by 6 h post treatment (Figure 7A,B). Expression of ER was observed in nuclear as well as cytoplasmic lysates of breast cancer cells treated with END and ENL within 15 min of treatment while E2-treated cells showed peak expression at later time points (Figure 7C). We questioned whether END and ENL treatment causes any change in the expression of canonical E2-responsive genes [74,75,76,77]. Indeed, breast cancer cells treated with END and ENL exhibited increased expression of E2-responsive genes (Figure 7D,E).

**Figure 8:**
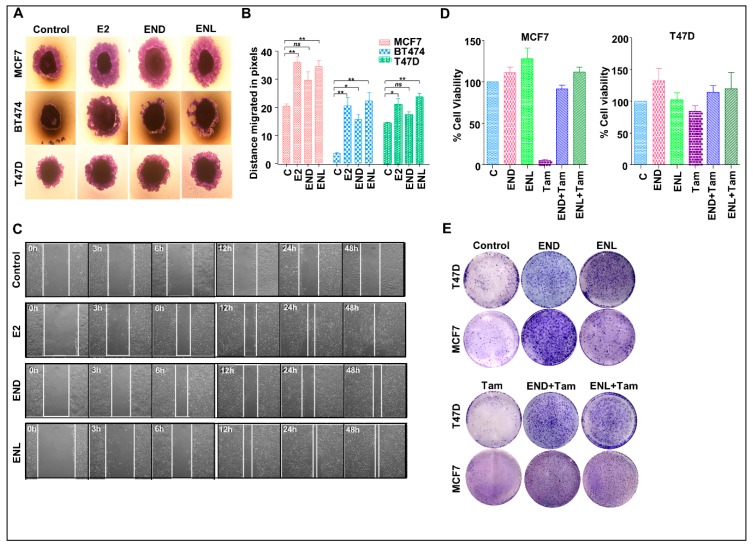
END and ENL induce migration and wound-healing in ER positive breast cancer cells. Pictorial (**A**) and graphical (**B**) representation of spheroid migration assay, MCF7, BT474, and T47D spheroids were formed on agarose followed by treatment with 1 μM END and 1 μM ENL. Statistically significant increase in rate of migration was observed. (**C**) MCF7 cells were treated with 1 μM END, 1 μM ENL and 100 nM E2 and wound healing was observed at regular intervals as indicated. (**D**,**E**) MCF7 and T47D cells were treated with 1 μM END, 1 μM ENL, 1 μM tamoxifen alone and in combination followed by cell viability and clonogenicity assay. END and ENL treatment reduced the effectiveness of tamoxifen. * *p* < 0.05; ** *p* < 0.005; ^ns^ *p* > 0.05.

To test the effect of END and ENL on the migration potential of breast cancer cells, we performed wound healing and spheroid migration assays (Figure 8A–C). Migration from MCF7, T47D and BT474 spheroids was significantly increased upon ENL treatment while END treatment only induced significant migration of BT474 cells (Figure 8A,B). While control MCF7 cells migrated at a rate of 0.52 pixcels/h, END induced 1.1875 pixcels/h migration while ENL resulted in 1.56 pixcels/h migration. The positive controls, E2 treated cells migrated at a rate of 1.45 pixcels/h (Figure 8B). Next, we sought to investigate whether END and/or ENL impact tamoxifen-mediated growth inhibition of breast cancer cells. Tamoxifen treatment inhibited cell viability and clonogenic growth of ER-positive breast cancer cells but cotreatment with END and ENL compromised the cytotoxicity of tamoxifen in MCF7 and T47D cell lines (Figure 8D,E). Collectively, these data suggest that END and ENL bind to ER and modulate ER-signaling cascade and increase breast cancer growth. END and ENL also interfere with the therapeutic efficacy of tamoxifen.

## 8. Conclusions

Most epidemiological studies have shown a protective effect of phytoestrogens and enterolignans against breast cancer especially in premenopausal women while a few studies have shown no effect or even negative effects. However, most of the studies estimate the levels of these active compounds based on the dietary consumption of plant-based foods but plant-based diets are rich source of multiple other active phytochemicals and health outcomes associated with plant-based diets cannot be attributed to enterolignans alone. For example, one study has shown that a flax seed rich diet in pregnant or lactating rats increases the risk of breast carcinogenesis in the progeny [78] and increased risk has been attributed to high cadmium levels [78]; no role of phytoestrogens or lignans has been described. Circulating enterolignans are solely dependent on the gut microbes that can vary based on ethnicity, BMI, geographical location, disease state, and drug/antibiotic usage. In totality, based on existing literature and our observations, we conclude that the enterolignans should neither be considered therapeutic against breast cancer nor as risk factor for developing breast cancer. Under normal physiological conditions in pre-menopausal women, estrogen levels are high enough to competitively inhibit binding of enterolignans to estrogen receptors. Even in individuals relying largely on plant based, lignan rich diet where enterolignans can reach very high levels, it is extremely unlikely that level of any one enterolignan would be selectively raised. It has already been determined from in vitro and in vivo studies that a combination of phytoestrogens like Ginseng and lignans nullify each other’s effect in the context of estrogen signaling [79,80]. Significant affect, might however, be observed in specific cases, e.g., in SIBO patients, extreme microbial activity may result in very high levels of enterolignans in the circulation; high enough to overcome E2′s competitive inhibitory effect. In post-menopausal women, where E2 reserves are depleted, it might prove detrimental. Similarly, high enterolignan levels might be protective against pregnancy associated breast cancers where they might compete with high circulating estrone for ligand binding site, thus inhibiting estrogen stimulation. The human microbiome is vast and dynamic; the symbiotic interaction between the genome and metagenome aids and complements various host physiological functions. It can promote or prevent breast cancers by producing genotoxins, steroid hormone metabolism, modulating immune response, regulating energy homeostasis and adiposity, regulating cytokine levels, so on and so forth. However, systemic studies demonstrating functional associations with specific taxonomic classes are limited. A thorough understanding of microbial signatures associated with breast cancers at both local tissue level as well as the GI tract and its functional implication is necessary to exploit the microbiome for preventing and treating breast cancers.

## Figures and Tables

**Table 1:** Differential abundance of gut microbial species in postmenopausal breast cancer patients reported by Zhu et al. [5].

Abundant in Postmenopausal Breast Cancer Patients	Abundant in Postmenopausal Breast Cancer Patients	Low Abundance in Postmenopausal Breast Cancer Patients
*Sodalis glossinidius*	*Escherichia coli*	*Eubacterium eligens*
*Escherichia_sp_TW11588*	*Shigella_sp_D9*	*Campylobacter concisus*
*Fusobacterium nucleatum*	*Escherichia_sp_3_2_53FAA*	*unclassified_Enterobacteriaceae_* *bacterium_9_2_54FAA*
*Shewanella putrefaciens*	*Shigella sonn*	*Roseburia inulinivorans*
*unclassified_Prevotella_sp._oral_taxon_299*	*Escherichia_sp_1_1_43*	*Brucella melitensis*
*unclassified Fusobacterium*	*Proteus mirabilis*	*Lactobacillus vaginalis*
*Yersinia enterocolitica*	*Shigella boydii*	*Escherichia albertii*
*Prevotella amnii*	*Vibrio cholerae*	
*Acidaminococcus intestine*	*Escherichia fergusonii*	
*Fusobacterium varium*	*Escherichia_sp_4_1_40B*	
*Acinetobacter radioresistens*	*Shigella flexneri*	
*Erwinia amylovora*	*Acinetobacter baumannii*	
*Salmonella enterica*	*Escherichia_sp_TW09276*	
*Enterococcus gallinarum*	*Actinomyces_sp_HPA0247*	
*Citrobacter koseri*	*Acinetobacter johnsonii*	
*Klebsiella_sp_1_1_55*	*Providencia rettgeri*	
*Desulfovibrio piger*	*unclassified_Citrobacter_sp._30_2*	
	*Citrobacter_sp_30_2*	

## Abbreviations

*E coli*	*Escherichia coli*
E1	estrone
E2	estradiol
E3	estriol
END	Enterodiol
ENL	Enterolactone
GI	Gastrointestinal
NSAID	Non-steroidal anti-inflammatory drugs
HSD	Hydroxysteroid dehydrogenases
BMI	Body mass index
OR	Odds ratio
HR	Hazard ratio
SIBO	Small intestinal bacterial overgrowth
LC/MS	Liquid chromatography/tandem mass spectrometry
EM	Estrogen metabolites
ERα	Estrogen receptor alpha
ERβ	Estrogen receptor beta
SBA	Secondary bile acids
AR	Androgen receptor
TNBC	Triple negative breast cancer
BRCA	BReast CAncer gene
